# A Novel Hybrid Machine Learning Based System to Classify Shoulder Implant Manufacturers

**DOI:** 10.3390/healthcare10030580

**Published:** 2022-03-20

**Authors:** Esra Sivari, Mehmet Serdar Güzel, Erkan Bostanci, Alok Mishra

**Affiliations:** 1 Computer Engineering Department, Cankiri Karatekin University, Cankiri 18100, Turkey; esrasivari@karatekin.edu.tr; 2 Computer Engineering Department, Ankara University, Ankara 06830, Turkey; mguzel@ankara.edu.tr (M.S.G.); ebostanci@ankara.edu.tr (E.B.); 3 Faculty of Logistics, Molde University College-Specialized University in Logistics, 6402 Molde, Norway; 4 Software Engineering Department, Atilim University, Ankara 06830, Turkey

**Keywords:** machine learning, hybrid models, shoulder implants, X-ray images

## Abstract

It is necessary to know the manufacturer and model of a previously implanted shoulder prosthesis before performing Total Shoulder Arthroplasty operations, which may need to be performed repeatedly in accordance with the need for repair or replacement. In cases where the patient’s previous records cannot be found, where the records are not clear, or the surgery was conducted abroad, the specialist should identify the implant manufacturer and model during preoperative X-ray controls. In this study, an auxiliary expert system is proposed for classifying manufacturers of shoulder implants on the basis of X-ray images that is automated, objective, and based on hybrid machine learning models. In the proposed system, ten different hybrid models consisting of a combination of deep learning and machine learning algorithms were created and statistically tested. According to the experimental results, an accuracy of 95.07% was achieved using the DenseNet201 + Logistic Regression model, one of the proposed hybrid machine learning models (*p* < 0.05). The proposed hybrid machine learning algorithms achieve the goal of low cost and high performance compared to other studies in the literature. The results lead the authors to believe that the proposed system could be used in hospitals as an automatic and objective system for assisting orthopedists in the rapid and effective determination of shoulder implant types before performing revision surgery.

## 1. Introduction

Shoulder implants vary according to their manufacturer and model. Shoulder implant surgery is performed on patients who have lost mobility due to shoulder injuries or arthritis. This invasive procedure, called Total Shoulder Arthroplasty (TSA), may need to be repeated in accordance the need to repair or replace the implant. In X-ray controls performed before implant repair or replacement surgery, specialists must predict the prosthesis manufacturer or model in cases where the patient has no previous records, where their records are not clear, or where the surgery was performed abroad. This situation makes it difficult for specialists in terms of time and cost. Distinguishing implant types and models for specific prosthesis manufacturers using an automatic expert system would be beneficial for both specialists and patients with respect to revision surgery operations.

In recent years, with the success of machine learning (ML) and deep learning (DL) algorithms in medical data processing applications, it has become vital to develop auxiliary expert systems that are able to save time in the decision-making process. Expert systems in medicine provide objective, cost-free, and high-speed diagnostic opportunities compared to medical diagnosis methods. In particular, with the development of algorithms such as convolutional neural networks (CNNs) [1], deep auto-encoders (DAEs) [2], and generative adversarial networks (GANs) [3] from DL methods, new methods in medical data processing have been discovered. The use of these methods compared to traditional algorithms in medical data processing has accelerated the application and calculation time of techniques such as feature extraction [4], classification [5], and segmentation [6].

In the field of orthopedics, ML and DL algorithms have been frequently used in studies such as the detection of fractures [7], the diagnosis and classification of osteoarthritis [8], the classification of arthroplasty implants [9,10], and the determination of bone age [11] on the basis of X-ray images, and very successful results have been achieved. In a recent study conducted by Lee and Chung in 2022 [12], the DL methods used for orthopedic diseases in medical image analysis were compiled. This study examined the classification of models and manufacturers of knee, hip, and shoulder arthroplasty implants from X-ray images. It was observed that relatively lower performance values were obtained in the classification of shoulder implants than in the classification of knee and hip implants. The authors interpreted the reason for this as spreading an anteroposterior shoulder X-ray over a wide range.

In a leading study conducted by Urban et al. in 2020 [13], a dataset containing 597 X-ray images with four manufacturer classes, Cofield, Depuy, Tornier, and Zimmer, was created to classify shoulder implant manufacturers. In the proposed classification method, six different convolutional neural networks (VGG-16, VGG-19, ResNet-50, ResNet-152, DenseNet, NASNet) and four different machine learning classifiers (Logistic Regression, Random Forests, Gradient Boost, and K-Nearest Neighbor) were used. When performing the classification, accuracy values between 74% and 80% were achieved using convolutional neural networks, while values between 51% and 56% were obtained with the other classifiers. This is was first study to classify shoulder implant manufacturers.

In another study, conducted by Yi et al. in 2020 [14], five different shoulder implant manufacturers (Biomet Bio-Modular: 37 images, DePuy Global: 125 images, DePuy HRP: 63 images, Stryker Solar: 51 images, Zimmer Bigliani-Flatow: 50 images) were classified by means of transfer learning with ResNet152. When performing the classification, AUC ROC values were obtained as follows: Biomet Bio-Modular, 0.95; DePuy Global, 0.95; DePuy HRP, 0.92; Stryker Solar, 0.86; and Zimmer Bigliani-Flatow, 1.0, distinguishing them from other classes.

Especially in recent years, the number of studies on the dataset created by Urban et al. [13] has increased. Various methods have been proposed for the classification of shoulder implant manufacturers. Vo et al. [15], in 2021, proposed a model called X-Net. The Squeeze and Excitation block integrated into the ResNet module was used in the X-Net model, and the X-Net model achieved 82% accuracy. Sultan et al. [16], in 2021, proposed the DRE-Net network, which consists of combining modified ResNet and DenseNet networks to divide shoulder implants into four classes. An accuracy of 85.92% was achieved with the DRE-Net network. Yılmaz [17], in 2021, proposed a deep learning model based on convolutional neural networks. In the proposed DL method, a new layer using a channel selection formula was applied to generate the filter properties. An accuracy of 97.2% was obtained with the proposed model. Zhou and Mo [18], in 2021, classified shoulder implants using the Random Forest, K-Nearest Neighbor, VGG16, ResNet50, InceptionV3, and Vision Transformer algorithms. With the 10-fold cross-validation ResNet50 network, 77% accuracy was achieved. Efeoğlu and Tuna [19], in 2021, selected Cofield, Depuy, and Zimmer classes from the same dataset and classified these three implant manufacturer types with 12 different machine learning algorithms. The highest values for accuracy were obtained using K-Nearest Neighbor, which achieved an accuracy of 100% without 10-fold cross-validation and 74% with 10-fold cross-validation. Karaci [20], in 2022, proposed convolutional neural network architectures pre-trained on the same dataset and cascade models consisting of the YOLOV3 algorithm. In the proposed method, after the shoulder implants had been detected with YOLOV3, the detected region was classified by pretrained convolutional neural networks. An accuracy of 84.76% was obtained with the YOLOV3 + DenseNet201 model.

In this study, two-stage hybrid ML algorithms using DL and ML algorithms are proposed and tested on the dataset created by Urban et al. [13] for the classification of shoulder implant manufacturers. This study aims to propose an automated, objective expert system application that can predict shoulder implant manufacturers. The proposed system can assist specialists before TSA surgeries in revision surgery. In a study conducted by Tang in 2013 [21], it was argued that when convolutional neural networks and SVM algorithms are used together, they are more successful than SoftMax. Later, this idea evolved into using neural networks with various ML classifiers. In the field of medical image processing, this method has been used in many studies, such as in the diagnosis of skin cancer [22], the classification of mammogram images [23], the early diagnosis of dementia [24], the diagnosis of COVID-19 disease [25], and the diagnosis of lung diseases [26], and successful results have been obtained. The hybrid methods proposed in this study are mainly inspired by a study conducted by Tang [21]. The difference is that the training phase is carried out using ML algorithms independently of DL algorithms. In our method, DL algorithms are used only for feature extraction. Constraints such as the inability of ML algorithms to work with raw data, and the inconvenience of traditional image processing techniques in feature extraction can be overcome by DL algorithms working with raw data and performing effortless feature extraction. Likewise, ML algorithms do not require hardware load in the learning phase, overcoming the high hardware cost constraints of DL algorithms. The proposed method for scientists working in this field provides an alternative and effortless solution to these disadvantages of DL and ML algorithms.

The main motivation behind the proposed study was to develop a new hybrid machine learning algorithm that is not as costly as DL frameworks to classify shoulder implants with high accuracy on the basis of X-ray images. The most important contribution of this study is that it presents a new hybrid method that can be applied to different classification problems. Although the success of DL algorithms on classification problems has been proven, this has led to the traditionalization of DL algorithms and the search for innovative methods. Another contribution of the study is to propose an expert system with high performance and low cost by using the proposed method to classify shoulder implant manufacturers.

Overall, the second section of this article presents the steps of the proposed method, the algorithms, applied ML techniques, and the dataset used in the application in a detailed way. On the other hand, the third section presents the results of the application, the discussion of the results, and a comparison with the literature. The article is then concluded by acknowledging the limitations of the study, outlining its contribution to science, and presenting suggestions for future work. The abbreviations frequently used in the rest of the article are given in Table 1.

## 2. Materials and Methods

### 2.1. Experimental Setup

All experiments in this study were performed on a laptop with Intel^®^ Core™ i7-9750H CPU, 16GB DDR4 RAM, and NVIDIA GeForce GTX 1660 Ti, GDDR6 6GB GPU. Application codes were written in Python using Keras [27] from DL libraries and Scikit-learn [28] from ML libraries.

### 2.2. Dataset

**Figure 1:**
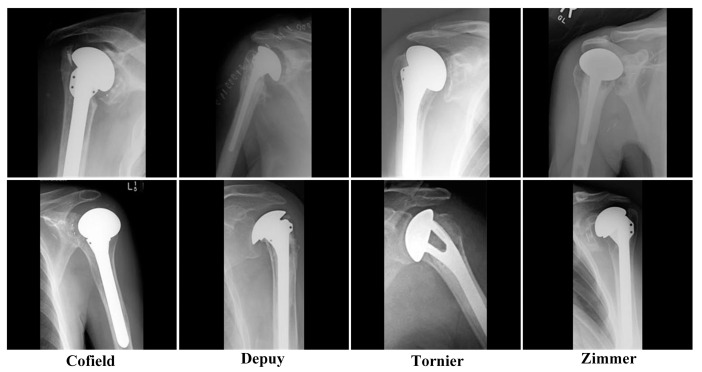
Samples from the dataset.

The dataset [13] used in the study includes 597 X-ray images of shoulder implants. In the dataset, there are X-ray images of 4 different shoulder implant manufacturers: 83 Cofield, 294 Depuy, 71 Tornier, 149 Zimmer. In Figure 1, X-ray samples of 4 classes in the dataset are given. Images are variable and have relatively low image resolution. The longest dimension of most of the images is 250 pixels. This dataset is publicly available, and all of the studies using this dataset will presented in Section 3.

When applying the proposed method, the dataset is divided into training, validation, and test sets. In Table 2, the number of images in each class is given according to the use of the dataset. Approximately 10% of the 597 X-ray images were used for the test set. A 5-fold cross-validation was applied, which allocated 11% of the validation set to the remaining 90% of the training set. The 10–11% rate was chosen when dividing the dataset into the test and validation set because the number of samples in the dataset is low, and the training set can be created with the maximum number of samples. Another reason is to keep the number of samples in test and validation sets approximately equal. While creating the test and validation set, sample selection was carried out using the Stratified Shuffle Split method for unbalanced datasets. Thus, random images were selected from each class in the percentages given. In addition, in each cross-validation fold, cases such as all the samples selected for validation entirely belong to a class or no samples from a class are prevented.

In the application, all images in the dataset were used in RGB (red, green, blue) mode. Pixel values were rescaled for normalization from 0–255 to 0–1. All images were resized to 224 × 224 with Bilinear interpolation algorithm [29] so that the computational load is compatible with the GPU and DenseNet201 architecture. Thus, all models used the same input size (224 × 224 × 3).

### 2.3. Proposed Method

The general structure of a convolutional neural network consists of a convolutional base and a classifier. The convolution base extracts public features in its first layers and class-specific features in its last layers. The classifier is added to the convolution base and classifies the features that come out of the convolution base. In the first step of the proposed method, the convolution base of a deep network model is run on the target dataset, and features are extracted from all images. Then the extracted features are saved to disk and given to the selected classifier as an input. This method greatly reduces the time and computational cost, as the convolutional base does not require training. However, in this method, the real-time data augmentation applied in DL algorithms cannot be applied to the training images, as the images will be passed through each layer only once. After the dataset is augmented, feature extraction can be used or a special algorithm that provides real-time data augmentation can be developed.

**Figure 2:**
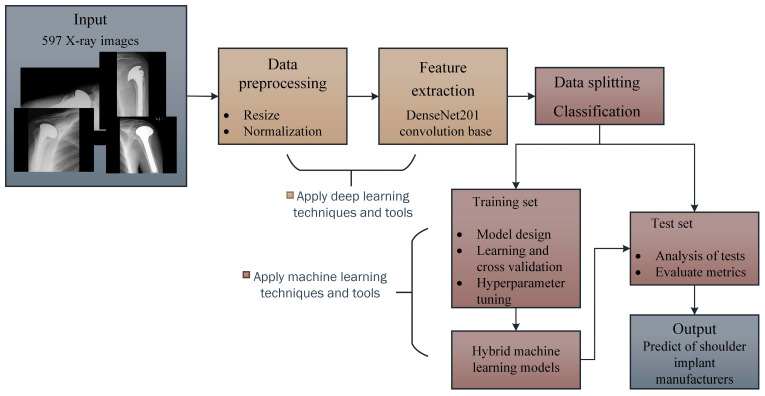
Overview of the proposed method.

A diagram showing the stages of the proposed method is given in Figure 2. DL techniques and tools were used in the first stage of the proposed two-stage hybrid method. After applying the data preprocessing techniques, each X-ray image in the dataset was passed through the convolution layer of the DenseNet201 [30] network, and the features were obtained. In the second stage, features were classified using ML techniques and tools. During the model design stage, the Logistic Regression (LR) [31], Linear Support Vector Machine (LSVM) [21], Multilayer Perceptron (MLP) [32], Linear Discriminant Analysis (LDA) [33], Random Forest (RF) [34], K-Nearest Neighbor (KNN) [35], Gaussian Naive Bayes (GNB) [36], Bernoulli Naive Bayes (BNB) [37], AdaBoost (AB) [38], and Decision Tree (DT) [39] algorithms were used. Models were passed through training, validation, and testing phases, and their results were evaluated.

### 2.4. Feature Extraction

The DenseNet201 architecture, designed by Huang et al. by training on the CIFAR, SVHN, and ImageNet datasets, used dense blocks where the output of all previous layers was combined [30]. Thanks to the use of dense blocks, each layer reuses the properties of all previous layers. Thus, feature propagation was strengthened, and the vanishing gradient problem was reduced. The use of a few filters in its architecture reduced the number of model parameters. DenseNet201 has the highest performance among pretrained DL networks with few parameters (ImageNet Top-5 accuracy: 93.6%). These advantages are the reason DenseNet201 was chosen as the feature extractor.

The feature was extracted, bypassing all the images in the dataset once from the convolution base of the DenseNet201 network given in Table 3. According to the shape of the final feature map (7 × 7 × 1920) of the DenseNet201 network, 94,080 numerical features were obtained from each image.

### 2.5. Classification

The GridSearchCV method was used for hyperparameter optimization in the modeling stage of the ML classifiers used in the application. In this method, hyperparameter and value combinations are tested one by one to find the models with the highest performance criteria. The ML classifiers of the Scikit-learn library used in the application and the values of their hyperparameters are given below.

- For the LR algorithm, multinomial LogisticRegression regularized in L2 norm [40] was used. The ‘newton-cg’ [41] optimization algorithm was used as the solver, and the c hyperparameter, which is the inverse of the regularization power, was taken as 0.1.
- For the LSVM algorithm, LinearSVC with ‘crammer_singer’ [42] optimization regularized in L2 norm was used, and regularization parameter c was taken as 1.0.
- For the MLP algorithm, MLPClassifier with 2 hidden layers, 100 neurons in the hidden layer, using Adam optimization algorithm [43] and ReLU activation function [44] was used. The learning rate was 0.001, the momentum coefficient was 0.8, and the epoch number was 200.
- For the LDA algorithm, LinearDiscriminantAnalysis with the SVD (Singular Value Decomposition) optimization algorithm [45], which is used for data with many features, was used.
- For the RF algorithm, RandomForestClassifier with 100 trees in the forest was used. The maximum depth number 500 as the stopping condition and Gini index [46] as the splitting quality criterion was taken.
- For the KNN algorithm, the KNeighborsClassifier with a K value of 38 using Manhattan distance was used.
- GaussianNB and BernoulliNB algorithms were used for the Gaussian (GNB) and Bernoulli (BNB) versions of the Naive Bayes algorithm.
- For the DT algorithm, the DecisionTreeClassifier with a maximum depth of 350 and the quality of division measured by the Gini criterion was used.
- For the AB algorithm, the AdaBoostClassifier, which uses 600 DT weak classifiers, was used.

**Figure 3:**
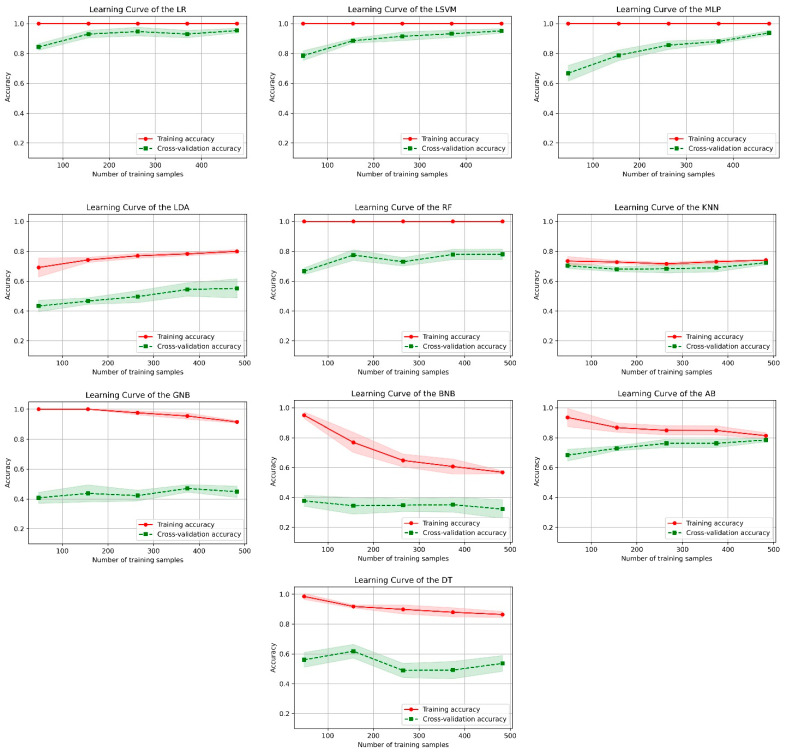
Learning curves of machine learning classifiers.

Five-fold cross-validation was applied to these classifiers during the training phase. During cross-validation, the training dataset is randomly divided into 5 separate sets at each iteration and used to make 4 training sets and 1 validation set. In Figure 3, the learning curves of the ML classifiers for the training phase are given. In the learning curves, the *x*-axis shows the accuracy of the algorithms, and the *y*-axis shows the amount of training data. In a learning curve of Figure 3, the red curve gives the training accuracy values, and the green curve gives the cross-validation accuracy values for the 5-fold cross-validation. When we examine the learning curves, the training accuracy score of the GNB, BNB, and AB algorithms decreased as the number of samples in the training set increased. This indicates that the generalization ability of these algorithms will not be affected even if there is more training data. According to the learning curves of other algorithms, adding more training samples will increase the generalization ability and the training and validation accuracy scores.

**Figure 4:**
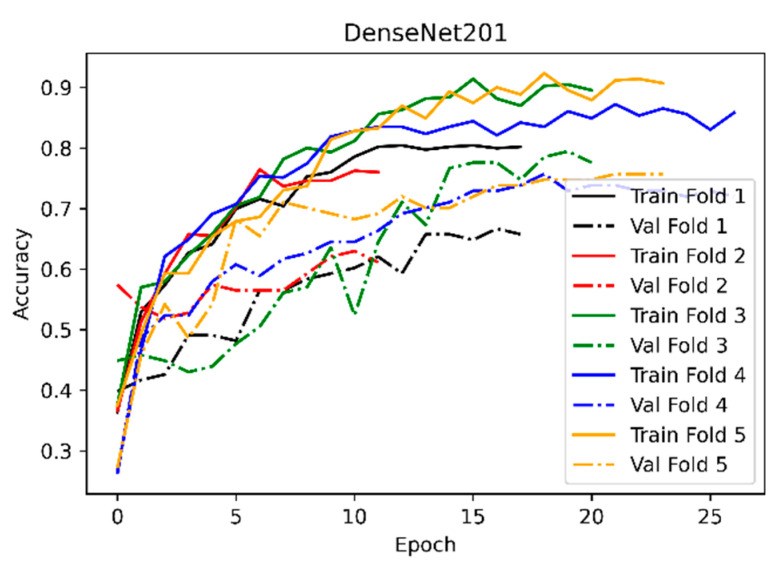
The learning curve of the DenseNet201 network.

To compare the scores of the hybrid ML algorithms used in the application according to the performance criteria with the SoftMax classifier, classification was also performed with the original DenseNet201 network. From the hyperparameters, weight initialization ‘imagenet’, loss function ‘categorical_crossentropy’, optimization algorithm Adam, learning rate 2 e-5, mini-batch size 8 were taken. In DenseNet201’s training, ‘imagenet’ weights were used only as initial weights. Since the ImageNet dataset and the shoulder implant manufacturer dataset were not similar, the DenseNet201 network was retrained, and weight freezing was not applied. During the training phase, 40-degree random rotation, 0.2 width–height shift, shear, and zoom were applied to the training set with real-time data augmentation. Points outside the bounds of the input were filled with the nearest mode. Five-fold cross-validation was applied with the training phase. The longest validation fold took 27 epochs, and the last validation fold took 24 epochs. In Figure 4, the learning curve of the DenseNet201 network, which shows the accuracy change according to the epoch change during the training phase, is given. The solid curves in Figure 4 represent the training accuracy, and the dashed curves represent the validation accuracy. Each of these curves provides the change in accuracy during a cross-validation fold. When examining the learning curve, it can be observed that the gaps between training and validation, namely the generalization gap, are too large for each fold. This situation is due to the small number of samples in the dataset, which increases the overfitting rate in the network.

## 3. Results and Discussion

### 3.1. Performance Metrics

**Figure 5:**
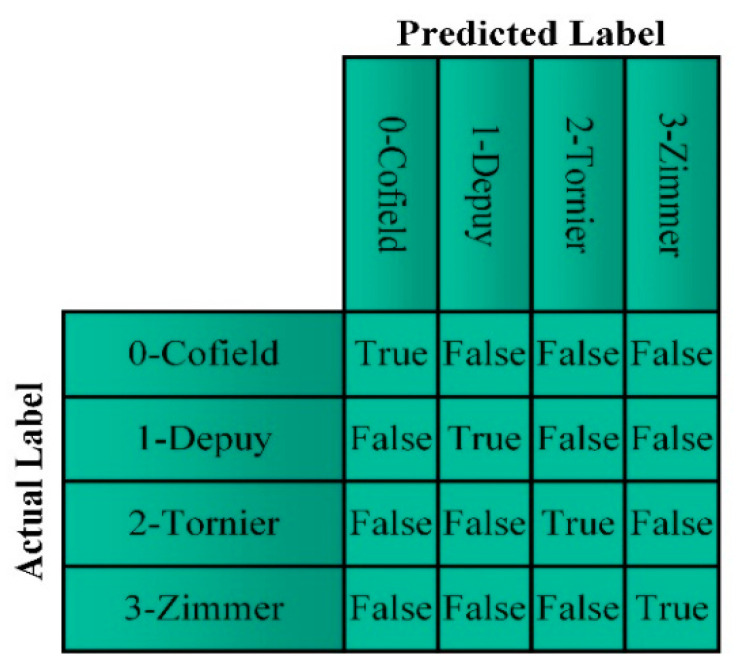
Confusion matrix.

The confusion matrix compares actual labels and labels predicted by the model as a number. In Figure 5, the meaning of the values given by a confusion matrix is shown. The confusion matrix provides the True Positive (TP), False Negative (FN), True Negative (TN), False Positive (FP) values used in the calculation of performance metrics.

Accuracy is the ratio of correct predictions to all predictions. Precision is the ratio of true positives to all positives. Recall measures how accurately the truly correct ones were predicted. The $F_{1}$ score gives the harmonic mean of precision and recall. In Table 4, mathematical formulas of accuracy, precision, recall, and $F_{1}$ score metrics are given. ROC is a probability curve with FPR (False Positive Rate) on the *X*-axis and TPR (True Positive Rate) on the *Y*-axis. The AUC value is obtained by calculating the area under the ROC curve, and it is desired that the AUC value be close to 1 for better discrimination.

As a result of the test phase, a 5 × 2 CV paired *t*-test [47] was applied to hybrid ML models whose accuracy values were close to each other. The 5 × 2 CV paired *t*-test procedure is used to compare the performances of the two algorithms. The dataset is divided into two—50% training and 50% test set—and training is repeated five times. As a result, t statistics and *p* values are obtained. The chosen significance level for the *p*-value was 0.05. A *p*-value less than 0.05 confirms the rejection of the null hypothesis; that is, there are significant differences between the two models.

### 3.2. Test Results and Discussion

**Figure 6:**
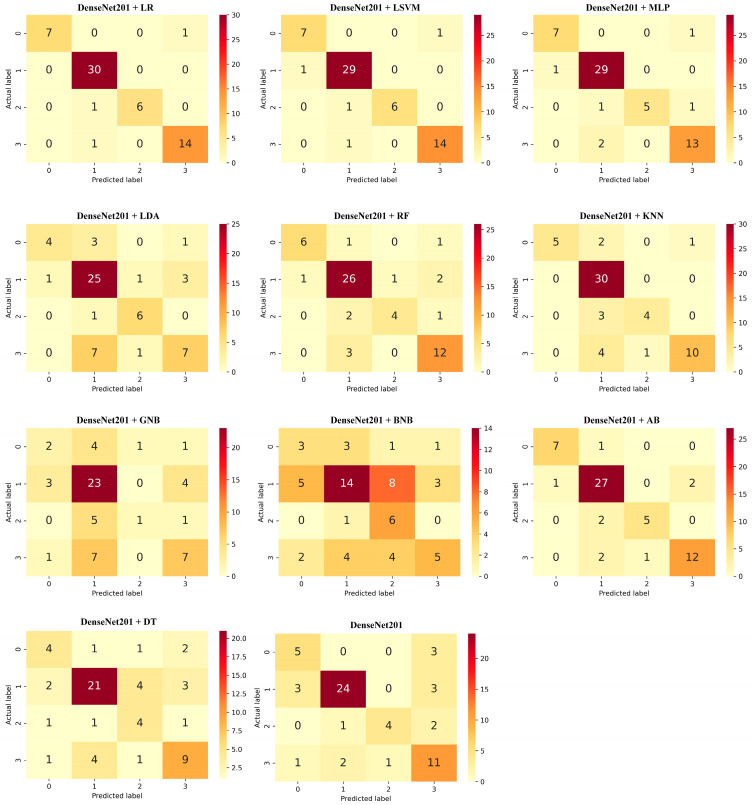
Confusion matrices of hybrid machine learning algorithms.

During the testing phase, 60 samples with the same preprocessing that the models had never seen were used. The confusion matrices of the proposed hybrid algorithms are shown in Figure 6; the representation as 0, 1, 2, and 3 present the Cofield, Depuy, Tornier, and Zimmer classes, respectively. Evaluation of confusion matrices alone is not sufficient to measure the success of algorithms on classes. For example, according to the confusion matrices given in Figure 6, DenseNet201 + LR, DenseNet201 + LSVM, DenseNet201 + AB and DenseNet201 + MLP algorithms classified the Cofield class with the highest number predictions (seven correct, one incorrect). Therefore, it is unclear which of these four algorithms distinguishes the Cofield from other classes class most successfully. In Table 5, the algorithms that made the highest number of predictions for each class according to the confusion matrices and the classification reports containing the precision, recall, and $F_{1}$ score values of these algorithms are given. According to the results in Table 5, the DenseNet201 + LR algorithm distinguished the Cofield, Depuy, Tornier, and Zimmer classes with the highest $F_{1}$ score values of 0.9333, 0.9677, 0.9231, 0.9333, respectively. The DenseNet201 + BNB algorithm is one of the algorithms that distinguishes the Tornier class with the greatest number of predictions according to the confusion matrix (six correct, one incorrect). In contrast, the DenseNet201 + BNB algorithm has a low $F_{1}$ score of 0.4615.

In Table 6, the test results obtained according to the selected performance metrics of the algorithms used in the application are given. The DenseNet201 + LR algorithm made the best classification, with 95.07% accuracy and 0.9677 macro average precision values. DenseNet201 + LR algorithm gave the highest values in all metrics compared to other algorithms. DenseNet201 + LSVM algorithm gave the second-highest result, with 93.31% accuracy and 0.9459 weighted average AUC ROC values. Among the high metric values, these algorithms are followed by DenseNet201 + MLP and DenseNet201 + AB algorithms, respectively. Considering the accuracy values, the DenseNet201 + RF algorithm seems more successful than the Dense-Net201 + KNN algorithm, but the Dense-Net201 + KNN algorithm’s macro average AUC ROC, $F_{1}$ score and weighted average AUC ROC values are higher than those of the DenseNet201 + RF algorithm. DenseNet201 + LDA, DenseNet201 + DT, DenseNet201 + GNB, and DenseNet201 + BNB algorithms gave accuracy values below 80%. The DenseNet201 + BNB algorithm has the lowest performance, with 46.67% accuracy and 0.5251 macro average AUC ROC values.

To verify the comparison of performance metrics made according to Table 6, a 5 × 2 CV paired *t*-test was applied to models with accuracy values close to each other. In Table 7, the pairs of algorithms for which the *t*-test was applied, and the results of this test are given. For the DenseNet201 + LR, DenseNet201 + LSVM, DenseNet201 + MLP and DenseNet201 + AB algorithms, which are the four algorithms that showed the highest accuracy, *p* < 0.05 in all *t*-test applications. This result shows that the performance evaluations made according to Table 6 for these four algorithms are accurate and reliable. As a result of the *t*-test between the DenseNet201 + RF and DenseNet201 + KNN algorithms, *p* > 0.05 shows that the performances of the two algorithms are the same. The fact that *p* > 0.05 for DenseNet201 + GNB and DenseNet201 + BNB algorithms shows no performance advantage against each other. This result shows that among the algorithms we experimented with, both DenseNet201 + GNB and DenseNet201 + BNB algorithms are the models with the lowest performance in the classification of shoulder implant manufacturers.

Although the DenseNet201 network gave higher results than most hybrid ML algorithms with 0.7333 accuracy and 0.7566 weighted average precision, it failed to beat DenseNet201 + LR, DenseNet201 + LSVM, DenseNet201 + MLP, DenseNet201 + AB, DenseNet201 + KNN and DenseNet201 + RF algorithms. This application yielded results that supported the study by Tang [21]. The mean fit time column given in Table 6 was recorded during the algorithms’ training and 5-fold cross-validation phases. The longest mean fit time in the proposed method is 18,1274 s with the DenseNet201 + AB algorithm. The mean fit time of the DenseNet201 algorithm is 83,4028 s. This result shows that the training time cost of the proposed method is quite low compared to DL algorithms.

In a study conducted by Zhou and Mo [18] on the same dataset, the dataset was augmented to increase the number of samples to 4173. In the dataset used without data augmentation, 53% validation accuracy was obtained with Random Forest and K-Nearest Neighbor. In the dataset using data augmentation, test accuracy values decreased to 48% and 41% for the same algorithms, respectively. The authors separated the training and test sets after augmenting the whole dataset in the study. It was clearly stated that the test set evaluated in the study mostly contained artificial samples. Real-time data augmentation during the training phase of DL algorithms does not carry the risk of artificial sample leakage into the validation set. However, in DL studies using the same dataset in the literature, data augmentation adversely affected the results. For example, in the study conducted by Urban et al. [13], NasNet trained with original data gave 80% validation accuracy, while NasNet trained with augmented data gave a validation accuracy value of 78.8%. In another study, conducted by Yılmaz [17] on the same dataset, the validation accuracy value of 97.20% in experiments with the original dataset decreased to 96.31% with the augmented dataset. The data augmentation applied to the training set should be proportional to the number of original samples. When the number of artificial samples obtained with a small and unbalanced dataset increases, overfitting is inevitable for the model.

In the application, 0.2 width–height shifts were applied to the training set, and 1908 images were obtained. Test accuracy results of hybrid ML algorithms with data augmentation are given in Table 8. When the accuracy values given in Table 8 are compared with the accuracy values given in Table 6, it can be observed that the test accuracy values decrease. The lower results of the data augmentation application that support the literature may be due to reasons such as the indiscriminate features obtained from the artificial samples and the number of artificial samples being higher than the original sample number. Data augmentation effects were not discussed in any study in the literature that classified shoulder implant manufacturers using the same dataset.

In Table 9, an overview of the studies conducted using the same dataset in the classification of shoulder implant manufacturers is given. When the proposed methods in these studies were examined, DL and ML algorithms were used for classification in all of the studies. However, in this study, a method in which DL and ML algorithms can be used in a hybrid form is proposed for the first time. In the study conducted by Urban et al. [13], accuracy values between 51 and 56% were obtained with the ML classifiers. In the study conducted by Zho and Mo [18], a 53% accuracy value was obtained with the ML algorithms. In the study conducted by Efeoğlu and Tuna [19], a 74% accuracy value was obtained with the KNN algorithm. In these studies, 10-fold cross-validation was performed. In our study, the DenseNet201 + KNN algorithm had an 80.11% test accuracy, while the equivalent DenseNet201 + RF algorithm had an 81.67% test accuracy. Six different algorithms were modeled using the method proposed in our study that presented test accuracy values above 80%. In our study, the test accuracy values of the DenseNet201 + GNB and DenseNet201 + BNB algorithms, which had the lowest performance values, were 55% and 46.67%, respectively. In general, as a result of these comparisons, it was observed that the hybrid ML algorithms proposed in this study gave better results than the results achieved in studies in which ML algorithms were used alone.

The results obtained in studies where DL algorithms were proposed are 80% and above [13,15,16,17,20]. In our study, the highest test accuracy values obtained with DenseNet201 + LR, DenseNet201 + LSVM, DenseNet201 + MLP and DenseNet201 + AB algorithms ranged from 96% to 85%. In addition to these algorithms, DenseNet201 + KNN and DenseNet201 + RF algorithms provided superior results compared to the method proposed by Zhou and Mo [18] with ResNet50. Apart from the accuracy of the method proposed by Yılmaz [17], these values outweigh the other methods proposed in the literature.

While the highest test accuracy value obtained in our study was 95.07%, the highest cross-validation accuracy value obtained by Yılmaz [17] was 97.20%. Our study contains significant and advantageous differences compared to the study conducted by Yılmaz [17]. In the study conducted by Yılmaz [17], the dataset was not divided into the test set and the validation set, and only the 5-fold cross-validation accuracy results were given in his study. The purpose of the validation dataset is to tune the model hyperparameters and measure the model’s capability. Since the validation dataset is used in the model configuration, evaluating the final results over the validation set will present the results in an optimistic light. Outcome evaluation should be carried out on an unbiased test set. In addition, no statistical test was applied to the results obtained in the study conducted by Yılmaz [17]. In our study, the evaluation of the final results was carried out on a separate test set from the cross-validation training and validation set. In addition, statistical tests were carried out to prove the real performance of the models in our study.

The most important advantage of the proposed method over DL algorithms is that it reduces time and hardware costs. The training time of the method proposed in the study conducted by Yılmaz [17] was 46,723 s, and the test time was 17,412 s. In our study, the DenseNet201 + AB model had the highest training time, with 18,1274 s. Training time is not important in developing and transferring this application to a real hospital environment, but a method with low time and hardware costs could help other scientists working in this field in different ways.

## 4. Conclusions and Future Directions

In this study, an expert system design for automatic and objective classification of shoulder implant manufacturers was presented. In the proposed method for this system, classification was carried out with 10 different hybrid ML algorithms in which DenseNet201 and ML algorithms were used together, as well as a DenseNet201 network trained by us, and the classification results were compared. Among the algorithms used, the DenseNet201 + LR algorithm predicted shoulder implant manufacturer classes with 95.07% accuracy, while the DenseNet201 network predicted with 73.33% accuracy. The proposed hybrid ML algorithms achieve the goal of low cost and high performance compared to other studies in the literature. This result indicates that the hybrid use of DL and ML algorithms can give better results compared to the use of DL or ML algorithms alone. This study has applicability in hospitals, as it offers a fast, objective, and automatic system that helps experts, alleviates the workload, and reduces costs.

A limitation of this study is the number of implant manufacturer classes in the dataset. Increasing the variety of implant manufacturers is an opportunity to expand the study. In addition, the small number of samples in the dataset greatly affected the high values of performance metrics. Changing the feature extractor DL algorithm, especially because of the small number of samples in the dataset, may increase the performance values by using networks with lower depths. Proposed hybrid algorithms should be developed by using a new dataset obtained by increasing the number of samples or diversifying feature extractor DL networks. In the proposed method, a feature selection algorithm can be developed in the feature extraction stage, and the selection of useful features for classification can increase the performance of the proposed method.

In future studies, masked implant images can be classified by obtaining masks of implants in X-ray images. Classification can be carried out on the augmented dataset using GANs. The proposed method in this study is applicable to all computer-aided diagnosis systems. The proposed feature extraction method is less costly than image processing methods, and the proposed classification method is less costly than training DL algorithms alone. These proposed method features can be beneficial to scientists working in this field. Such applications can be integrated into medical devices, computers, and mobile applications in the future.

## Figures and Tables

**Table 1:** Abbreviations.

Abbreviation	Explanation
AB	AdaBoost
BNB	Bernoulli Naive Bayes
DL	Deep Learning
DT	Decision Tree
GNB	Gaussian Naive Bayes
KNN	K-Nearest Neighbor
LDA	Linear Discriminant Analysis
LR	Logistic Regression
LSVM	Linear Support Vector Machine
ML	Machine Learning
MLP	Multilayer Perceptron
RF	Random Forest
CV	Cross-Validation

**Table 2:** Use of the dataset in the application.

	Training	Test
Cofield	75	8
Depuy	264	30
Tornier	64	7
Zimmer	134	15
Total	537	60

**Table 3:** The convolutional base of the DenseNet201 network.

Layers	Output Size	Operation
Conv	112 × 112	7 × 7 conv, stride 2
Pooling	56 × 56	3 × 3 max-pool, stride 2
DB1	56 × 56	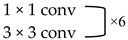
TL1	56 × 56	1 × 1 conv
	28 × 28	2 × 2 avg-pool, stride 2
DB2	28 × 28	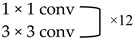
TL2	28 × 28	1× 1 conv
	14 × 14	2 × 2 avg-pool, stride 2
DB3	14 × 14	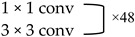
TL3	14 × 14	1 × 1 conv
	7 × 7	2 × 2 avg-pool, stride 2
DB4	7 × 7	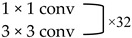

Input size: 224 × 224 × 3; Conv: Convolution layer; DB: Dense block; TL: Transition layer; conv: Convolution; max-pool: Maximum pooling; avg-pool: Average pooling.

**Table 4:** Performance metrics.

Performance Metrics	Mathematical Formulas
Accuracy	$\frac{TP+TN}{TP+FP+FN+TN}$
Precision	$\frac{TP}{TP+FP}$
Recall	$\frac{TP}{TP+FN}$
$F_{1}$ Score	$2\;\times \;\frac{Precision\;\times \;Recall}{Precision\;+\;Recall}$

*TP*: True Positive; T*N*: True Negative; F*P*: False Positive; F*N*: False Negative.

**Table 5:** Classification reports of algorithms making the highest numbers of predictions based on the confusion matrix.

Class	Prediction Numbers	Algorithm	Precision	Recall	$F_{1}$ Score
Cofield (0)	7 correct 1 incorrect	DenseNet201 + LR	1.0000	0.8750	0.9333
		DenseNet201 + LSVM	0.8741	0.8750	0.8745
		DenseNet201 + AB	0.8738	0.8749	0.8743
		DenseNet201 + MLP	0.8746	0.8740	0.8742
Depuy (1)	30 correct 0 incorrect	DenseNet201 + LR	0.9375	1.0000	0.9677
		DenseNet201 + KNN	0.8125	0.8667	0.8387
Tornier (2)	6 correct 1 incorrect	DenseNet201 + LR	1.0000	0.8571	0.9231
		DenseNet201 + LSVM	1.0000	0.8568	0.9228
		DenseNet201 + LDA	0.7500	0.8571	0.8000
		DenseNet201 + BNB	0.3158	0.8571	0.4615
Zimmer (3)	14 correct 1 incorrect	DenseNet201 + LR	0.9333	0.9333	0.9333
		DenseNet201 + LSVM	0.9321	0.9333	0.9326

**Table 6:** Test results of algorithms used in the application.

			Macro Average				Weighted Average			
Algorithm	Mean Fit Time (s)	Accuracy (%)	Precision	Recall	$F_{1}$ Score	AUC	Precision	Recall	$F_{1}$ Score	AUC
DenseNet201 + LR	10.2057	95.07	0.9677	0.9164	0.9394	0.9471	0.9521	0.9500	0.9493	0.9556
DenseNet201 + LSVM	10.1041	93.31	0.9360	0.9080	0.9206	0.9405	0.9344	0.9333	0.9331	0.9459
DenseNet201	83.4028	73.33	0.7058	0.6824	0.6860	0.7423	0.7566	0.7333	0.7390	0.7469
DenseNet201 + MLP	7.8994	89.62	0.9120	0.8557	0.8776	0.9074	0.9031	0.9000	0.8983	0.9182
DenseNet201 + LDA	3.1186	70	0.7202	0.6643	0.6779	0.7843	0.7005	0.7000	0.6888	0.7911
DenseNet201 + RF	2.1407	81.67	0.8696	0.7158	0.7687	0.8153	0.8386	0.8167	0.8074	0.8295
DenseNet201 + KNN	0.3374	80.11	0.8049	0.7470	0.7699	0.8326	0.8014	0.8000	0.7973	0.8365
DenseNet201 + GNB	0.5175	55	0.4904	0.4065	0.4187	0.5645	0.5323	0.5500	0.5224	0.5663
DenseNet201 + BNB	0.5688	46.67	0.4519	0.5080	0.4375	0.5251	0.5339	0.4667	0.4717	0.5386
DenseNet201 + AB	18.1274	85.63	0.8523	0.8223	0.8357	0.8800	0.8500	0.8500	0.8488	0.8754
DenseNet201 + DT	7.5126	63.33	0.5694	0.5929	0.5769	0.7310	0.6522	0.6333	0.6400	0.7383

**Table 7:** 5 × 2 CV paired *t*-test results.

Algorithms	t Statistic	*p*-Value
DenseNet201 + LR − DenseNet201 + LSVM	3.530	0.017
DenseNet201 + LR − DenseNet201 + MLP	2.896	0.034
DenseNet201 + LR − DenseNet201 + AB	15.403	0.000
DenseNet201 + LSVM − DenseNet201 + MLP	2.778	0.039
DenseNet201 + LSVM − DenseNet201 + AB	8.696	0.000
DenseNet201 + MLP − DenseNet201 + AB	−3.900	0.011
DenseNet201 + RF − Dense-Net201 + KNN	−0.204	0.846
DenseNet201 + LDA − DenseNet201 + DT	−2.743	0.041
DenseNet201 + GNB − DenseNet201 + BNB	1.388	0.224

**Table 8:** Test accuracy results with data augmentation.

Algorithm	Accuracy (%)
DenseNet201 + LR	92.07
DenseNet201 + LSVM	89.36
DenseNet201 + MLP	82.57
DenseNet201 + LDA	68.57
DenseNet201 + RF	74.61
DenseNet201 + KNN	69.60
DenseNet201 + GNB	51.08
DenseNet201 + BNB	45.39
DenseNet201 + AB	74.49
DenseNet201 + DT	53.90

**Table 9:** Overview of studies conducted using the same dataset.

Author, Year, Reference	Classes	Dataset	Method	Results Accuracy %
Urban et al., 2020, [13]	Cofield, Depuy, Tornier, Zimmer	All 4-classes dataset: 597	6 different DL algorithms: VGG-16, VGG-19, ResNet-50, ResNet-152, DenseNet, and NASNet and 4 different ML algorithms: Logistic Regression, Random Forests, Gradient Boost, and K-Nearest Neighbor; 10-fold cross-validation	DL algorithms: 74–80%; ML algorithms: 51–56%
Vo et al., 2021, [15]	Cofield, Depuy, Tornier, Zimmer	All 4-classes dataset: 597	X-Net: Squeeze and Excitation block integrated into the ResNet module; 10-fold cross-validation	82%
Sultan et al., 2021, [16]	Cofield, Depuy, Tornier, Zimmer	All 4-classes dataset: 597	DRE-Net: Combination of modified ResNet and DenseNet; 10-fold cross-validation	85.92%
Yılmaz, 2021, [17]	Cofield, Depuy, Tornier, Zimmer	All 4-classes dataset: 597	DL network with a new layer using a channel selection formula; 5-fold cross-validation	97.2%
Zhou and Mo, 2021, [18]	Cofield, Depuy, Tornier, Zimmer	All 4-classes dataset: 597	Random Forest, K-Nearest Neighbor, VGG16, ResNet50, InceptionV3 and Vision Transformer algorithms; 10-fold cross-validation	ResNet50: 77%
Efeoğlu and Tuna, [19]	Cofield, Depuy, Zimmer	All 3-classes dataset: 349	12 different ML algorithms; 10-fold cross-validation	K-Nearest Neighbor: 74%
Karaci, 2022, [20]	Cofield, Depuy, Tornier, Zimmer	All 4-classes dataset: 597	Cascade models consisting of pretrained convolutional neural network architectures and the YOLOV3 algorithm; 10-fold cross-validation	YOLOV3 + DenseNet201: 84.76%
This study	Cofield, Depuy, Tornier, Zimmer	All 4-classes dataset: 597	Hybrid ML algorithms; 5-fold cross-validation	DenseNet201 + Logistic Regression: 95.07%

## Data Availability

Not applicable.
